# Synergistic Effect of Retinoic Acid Polymeric Micelles and Prodrug for the Pharmacodynamic Evaluation of Tumor Suppression

**DOI:** 10.3389/fphar.2019.00447

**Published:** 2019-05-16

**Authors:** Yan-Hua Zhu, Ning Ye, Xin-Feng Tang, Malik Ihsanullah Khan, Hong-Liang Liu, Ning Shi, Li-Feng Hang

**Affiliations:** ^1^School of Life Sciences, University of Science and Technology of China, Hefei, China; ^2^Shandong Liangfu Pharmaceutical Co., Ltd., Jining, China

**Keywords:** all-trans retinoic acid, Pluronic F127, cisplatin, breast cancer, combination

## Abstract

All-trans retinoic acid (ATRA) is an effective agent that induces differentiation, inhibits cell proliferation, and acts as an anticancer agent. ATRA was successfully conjugated with Pluronic F127 via esterification to enhance its anticancer effects. Pluronic-ATRA showed high cytotoxicity and inhibitory concentrations (IC_50_) 50% lower than those of ATRA in various breast cancer cell lines (4T1:31.16–8.57 μg/mL; EMT6: 50.48–7.08 μg/mL; MDA-MB-231:37.58–8.99 μg/mL; BT474:25.27–9.09 μg/mL). In combination with chemotherapy, Pluronic-ATRA synergistically enhanced the cytotoxic effects of cisplatin (CDDP). Pluronic-ATRA combined with CDDP effectively suppressed breast tumor growth *in vivo*. The results of this study demonstrate the potential of Pluronic-ATRA as an anticancer agent that can be used in combination therapy against solid tumors.

## Introduction

Breast cancer results in the development of destructive tumors and leads to the highest rate of cancer-related deaths in females around the world (Wang et al., 2017; Zhang et al., 2017). Breast cancer therapy is currently based on clinical and pathological findings but shows limited therapeutic efficacy and is restricted to sequential chemotherapy, radiation, and surgery (Misra et al., 2010). Cisplatin (CDDP) is generally used as a first-line therapeutic agent against blood cancer (lymphoma and myeloma) and solid tumors (small cell lung, non-small cell lung, ovarian, stomach, bladder, and particularly testicular cancers) because of its ability to cross-link with DNA after entering the cancer cell (Wang and Lippard, 2005). CDDP binds DNA double-helix strands at atom N7 of guanine bases, thereby impeding double strands from uncoiling and separating. This prevents cell division and leads to programmed cell death and apoptosis (Pasini and Zunino, 1987; Galanski et al., 2003; Aryal et al., 2009). Replacing cytotoxic drugs with combinations (Chen et al., 2017a; Zhang et al., 2018) involving other therapeutic agents such as photosensitizers and neovasculature disruption agents as well as traditional drugs represents an effective alternative to cancer therapy (Li et al., 2017). Combinations of two or more therapeutic agents or strategies present unique advantages, including the inhibition of different signaling pathways, target-specific actions, improved drug efficacy, and reduced off-target toxicity (Bang et al., 2010).

Retinoic acid (RA) is a natural derivative of vitamin A whose anticancer activity has received much attention (Zhu et al., 1997; Siddikuzzaman et al., 2011). Although RA does not generally inhibit cell growth, it has been proposed that it suppresses tumor growth by inducing cell differentiation, inhibiting cell proliferation, and exerting anti-migration and -invasion effects on tumor cells (Siddikuzzaman et al., 2011). RA has different natural and synthetic compound derivatives called retinoids, which affect cell differentiation, growth, and apoptosis. Retinoids have the ability to induce differentiation and apoptosis in cancer cells via cytotoxic and anti-oxidant activities, suggesting their potential as chemotherapeutic agents against cancer (Chen et al., 2014). All-trans retinoic acid (ATRA), the most abundant natural analog, displays activity against various cancers such as lymphoma, leukemia, neuroblastoma, and lung, cervical, and kidney cancers, among others. ATRA controls cancer growth via unique mechanisms, such as the induction of cell differentiation and anti-proliferation, -migration, and -invasion effects (Zhu et al., 1997; Park et al., 2011; Siddikuzzaman et al., 2011; Chen et al., 2014). ATRA suppressed the growth of human breast cancer cells (MCF-7) *in vitro* when used at concentrations >10 nM. Other studies have shown that ATRA inhibited the growth of hepatocellular carcinoma (Zhu et al., 1997). Clinical studies also showed the chemo-preventive effects of RA: ATRA, used as adjutant, effectively suppressed the occurrence of secondary cancers in patients with early-stage skin, head and neck, breast, and hepatocellular cancers (Sano et al., 2003; Clerici et al., 2004; Khuri et al., 2006). Certain studies also reported the synergistic therapeutic effects of ATRA and its derivatives in combination with chemotherapeutics such as doxorubicin, CDDP, and paclitaxel in inducing receptor-mediated cytotoxicity and inhibiting cell growth factors (Liu et al., 2012; Yao et al., 2013; Sun et al., 2015). Therefore, the use of RA in combination with chemotherapeutic agents contributes to enhancing the therapeutic efficacy and decreasing side effects. However, the clinical applications of ATRA are limited by low water solubility and plasma concentrations, systemic side effects (such as acute retinoid resistance, mucocutaneous dryness, hypertriglyceridemia, and headache), physicochemical instability, and few effective delivery systems (Emami et al., 2018; Wang et al., 2018a; Yu et al., 2019).

Nanotechnology-based medicines have shown significant advantages for cancer treatment in the last two decades, thus improving the shortcomings of current therapeutic agents by prolonging drug exposure through blood circulation, improving pharmacokinetic parameters, and enhancing tumor accumulation and cellular uptake (Peer et al., 2007; Davis et al., 2010a; Doane and Burda, 2012; Yuan et al., 2012). Nanomedicine improves therapeutic agent efficiency in cases of cancer treatment limitations including drug resistance, off-target effects, and metastasis by ensuring targeted and multimode efficacy (Davis et al., 2010b; Meng et al., 2010; Sarfati et al., 2011; Yang et al., 2014). Nano-based delivery systems, including liposomal (Ozpolat et al., 2003), polymeric (Cho et al., 2001; Sun et al., 2014; Chen et al., 2015; Xiao et al., 2018), and lipid nanoparticles (Lim and Kim, 2002; Castro et al., 2007), have been used to deliver RA to cancer cells. However, the RA loading rate remains mostly low and unstable, easily resolving during storage or when entering the blood circulation after injection. In a recent study, new hybrid nanoparticles composed of polymer-oil-based nano-carriers were used to stabilize RA and increase its stability during delivery (Narvekar et al., 2012). Although this new delivery system showed more effective anti-cancer activity compared to that of free RA, its preparation is complicated by low solubility, and these nanoparticles cannot be used for co-delivery of RA with other lipophilic drugs. In other studies, RA was conjugated to different polymers to increase loading efficiency and control the RA release rate (Hou et al., 2012; Zhang et al., 2015; Cao et al., 2018). For example, chitosan oligosaccharide was conjugated with RA to prepare polymer nanoparticles for co-delivery of RA and paclitaxel (Zhang et al., 2015). However, RA-conjugated nanoparticles did not display effective *in vitro* cytotoxicity because of the low degree of RA conjugation.

In the current study, we developed a polymeric micelle (Feng et al., 2017; Wang et al., 2018b) by conjugating ATRA to Pluronic F127 to form mixed micelles for efficient delivery. Pluronics are polymers composed of poly(ethylene oxide)-poly(propylene oxide)-poly(ethylene oxide) (PEO-PPO-PEO), in which PPO is hydrophobic and PEO is a hydrophilic (Moebus et al., 2009). Of all the Pluronic agents, Pluronic F127 is widely considered the most appropriate for biomedical applications (Akash and Rehman, 2015). Pluronic F127 has the ability to provide a hydrophobic core (Chen et al., 2017b) structure in aqueous solutions, inside which hydrophobic drugs such as ATRA can be solubilized and stabilized, so the low loading efficacy and instability of ATRA was overcome in this study by forming pluronic-ATRA micelles. Moreover, the combination of ATRA and CDDP effectively inhibited cancer cell proliferation and suppressed tumor growth *in vivo*. This work focused on the synthesis of F127-ATRA nanoparticles and the cytotoxic assessment of F127-ATRA micelles on different breast cancer cell lines *in vitro*. Furthermore, we tested whether low-dose F127-ATRA micelles and the traditional chemotherapeutic agent CDDP showed synergistic cytotoxic effects against breast carcinoma both *in vitro* and *in vivo*.

## Materials and Methods

### Materials

Pluronic F127 and ATRA were obtained from Sigma-Aldrich (St. Louis, MO, United States) and CDDP was purchased from Shandong Boyuan Pharmaceutical Co., Ltd. (Jinan, China). The 1-(3-Dimethylaminopropyl)-3-ethylcarbodiimide hydrochloride (EDC) and 4-(dimethylamine)-pyridine (DMAP) were purchased from Aladdin Industrial Co., Ltd. (Ontario, CA, United States). Dichloromethane (DCM) was distilled under reduced pressure before use. Water was ultra-purified using a Milli-Q water system (Millipore, Burlington, MA, United States) consisting of a carbon filter cartridge, two ion-exchange filter cartridges, and an organic removal cartridge. All other solvents and reagents were used as received unless specified.

### Synthesis of Pluronic-ATRA

Pluronic-ATRA was synthesized via esterification. ATRA (120 mg), Pluronic (1000 mg), EDC⋅HCl (115.2 mg), and DMAP (24.4 mg) were mixed in 10 mL DCM. The mixture was stirred in the dark for 48 h at 20°C. Excess ATRA was removed by precipitation in ice-cold ether. To completely remove free EDC⋅HCl and DMAP, the solution was transferred to a dialysis membrane (Spectra/Por^®^, Float-A-Lyzer^®^, molecular weight cut-off (MWCO) = 1 kDa, Sigma-Aldrich) and dialyzed against ultra-purified water three times for 24 h at 4°C, before further drying using a freeze dryer (Labconco, Kansas City, MO, United States) to completely remove the water. The final product Pluronic-RA was a yellow powder with a percentage grafting of 83%. The product was analyzed via nuclear magnetic resonance (NMR) and Fourier-transform infrared (FTIR) spectroscopy.

### Cell Culture

The breast cancer cell lines 4T1, MDA-MB-231, EMT6, and BT474 were purchased from the American Type Culture Collection (ATCC, Manassas, VA, United States) and cultured in Dulbecco’s modified Eagle’s medium (DMEM, Gibco, Thermo Fisher Scientific, Waltham, MA, United States) supplemented with 10% fetal bovine serum (FBS, ExCell Bio, Shanghai, China) and 1% penicillin/streptomycin (Gibco). All cells were incubated at 37°C in a humidified atmosphere containing 5% CO_2_ and were harvested via centrifugation at 800 ×*g* for 5 min to stimulate propagation.

### *In vitro* Cytotoxicity Assays

For the toxicity study, MDA-MB-231, BT474, 4T1, and EMT6 cells (5 × 10^3^) in 100 μL DMEM supplemented with 10% FBS were seeded in 96-well plates and incubated for 24 h at 37°C in a 5% CO_2_ atmosphere. The next day, the culture medium was removed and 100 μL fresh DMEM containing serial dilutions of ARTA, Pluronic F127, and Pluronic-ATRA (in triplicates). After an additional 48 or 72 h incubation, 20 μL 3-[4,5-dimethylthiazol-2-yl]-2,5 diphenyl tetrazolium (MTT) solution (5 mg/mL) was added to each well. After further incubation for an additional 4 h, the MTT medium was replaced with 150 μL extraction buffer [dimethyl sulfoxide (DMSO) at 20°C] before gently tapping the 96-well plate to dissolve the dye and incubating for 10 min at 37°C. The absorbance of the each well was measured at 490 nm using a Bio-Rad 680 microplate reader (Bio-Rad, Hercules, CA, United States). Cell viability was normalized to that of the untreated control group, which served as an indicator of 100% cell viability.

### Combination of Pluronic-ATRA and CDDP

MDA-MB-231 and BT474 cells (2 × 10^3^) as well as 4T1 and EMT6 cells (1 × 10^3^) were seeded in 96-well plates in 100 μL DMEM and incubated at 37°C in a 5% CO_2_ atmosphere for 24 h. Cells were then divided into three groups: two pretreatment groups and a non-pretreatment group. For the pretreatment groups, the culture medium was replaced by 100 μL fresh medium containing Pluronic-ATRA (10^-5^ M). After 48 h, one of the pretreatment groups was treated with CDDP in 100 μL DMEM and the other group was treated with Pluronic-ATRA (10^-5^ M) and CDDP in 100 μL DMEM and incubated for an additional 72 h. For the non-pretreatment group, the cells were treated with CDDP alone for 72 h. Next, 100 μL fresh medium (containing 20 μL 5 mg/mL MTT stock solution in Milli-Q Water) was substituted for the old medium, with the exception of the wells used as blanks, to which the same volume of PBS was added and cultured for a further 4 h. After this, 150 μL extraction buffer (DMSO at 37°C) was added to the wells and incubated for 10 min at 37°C. The absorbance of the solution was measured at 490 nm using a Bio-Rad 680 microplate reader (Bio-Rad), and cell viability was normalized to that of cells cultured in culture medium with PBS treatment.

### Xenograft Tumor Model

Female mice (6 weeks old) were purchased from the Vital River Laboratory Animal Technology Co., Ltd. (Beijing, China). All animals received care provided according to the regulations of animal care at the University of Science and Technology of China (USTC, Hefei, China). The study was approved by the USTC Animal Care and Use Committee. All animal experimental protocols conformed to the guidelines outlined in the Guide for the Care and Use of Laboratory Animals by the Laboratory Animal Center in USTC.

The different breast tumor xenograft models were generated using EMT6 cells (1 × 10^6^) with 20% Matrigel (BD Biosciences, Franklin Lakes, NJ, United States) in 100 μL PBS injected into the mammary fat pat of female BALB/c mice. Tumor-bearing mice were used when the volume of tumors reached 80–100 mm^3^.

### *In vivo* Tumor Suppression Evaluation

When the tumor reached a volume of 80–100 mm^3^, mice were randomly divided into four groups (*n* = 4 per group). Mice in the four groups were treated with PBS (control), Pluronic-ATRA (3 mg/kg), CDDP (2 mg/kg), or Pluronic-ATRA (3 mg/kg)/CDDP (2 mg/kg) via intravenous (i.v.) injection every 3 days, respectively. The volume of the tumor was measured using a venire caliper every second day and the volume was calculated according to the following formula:

$$volume=\frac{1}{2}\times length\times width^{2}$$

Changes in the body weights of mice were determined using an electronic scale. At the end of the *in vivo* therapeutic efficacy study, the mice were sacrificed and tumor tissues were harvested, weighed, and photographed.

### Statistical Analysis

Statistical significance was assessed via one-way analysis of variance (ANOVA) using SPSS Statistics software (Version 22.0, SPSS Inc., Chicago, IL, United States). *P*-values <0.05 were considered to indicate statistical significance (95% confidence level).

## Results

### Synthesis and Characterization of Pluronic-ATRA

**FIGURE 1:**
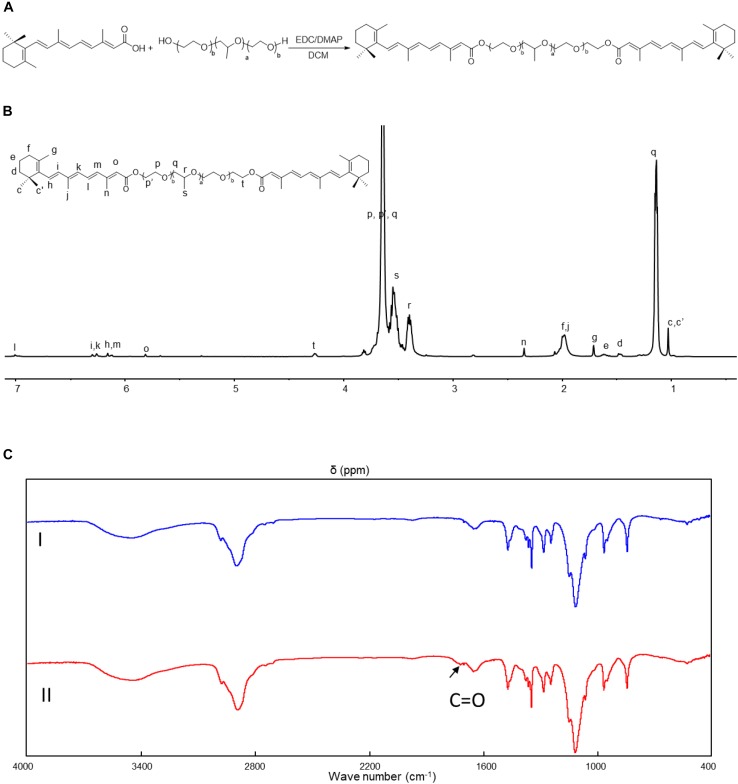
**(A)** Schematic illustration for the synthesis of Pluronic-retinoic acid (RA), **(B)** ^1^H nuclear magnetic resonance (NMR) spectrum of Pluronic-all-trans RA (ATRA) (in CDCl_3_, ppm), **(C)** Fourier-transform infrared (IR) spectrum of Pluronic-ATRA.

The synthesis of Pluronic-ATRA is depicted in Figure 1A. In this study, Pluronic-ATRA was synthesized through a condensation reaction. The reaction was conducted in DCM at 20°C with EDC/DMAP as the catalyst. The catalyst is advantageous as it can be used in small doses and can react at 20°C. The yield was approximately 80%. The structure of the final conjugation was confirmed via ^1^H NMR and FTIR spectroscopy (Figure 1B,C).

The ^1^H NMR spectrum of Pluronic-ATRA is shown in Figure 1B. The following resonances were assigned to the ^1^H NMR spectrum (CDCl3, 400 MHz) : δ 1.02 (24H, c, c’), 1.06–1.22 (12H, q), 1.46 (4H, d), 1.61 (4H, e), 1.97 (4H, f), 1.71 (6H, g), 6.10–6.18 (4H, h, m), 6.24–6.32 (4H, I, k), 1.98 (6H, j), 7.0 (2H, l), 2.35 (6H, n), 5.81 (2H, o), 3.58–3.84 (516H, p, p’, q), 3.30–3.43 (56H, r), 3.43–3.59 (168H, s), 4.26 (4H, t) ppm.

As shown in Figure 1C, the chemical structure of Pluronic-ATRA was further confirmed via FTIR spectroscopy. The presence of the strong absorption bands at 1700 and 1626 cm^-1^ (Figure 1C, II) for the newly formed ester C = O moieties is further proof of the successful synthesis of the desired conjugate, thus confirming the presence of newly attached groups to F127, which were not seen in Figure 1C, I. The Pluronic-ATRA micelles were formed by the traditional strategy, and the average size of micelles is approximate 30 ± 2 nm (Supplementary Figure S1).

### *In vitro* Cytotoxicity of ATRA in Breast Cancer Cells

**FIGURE 2:**
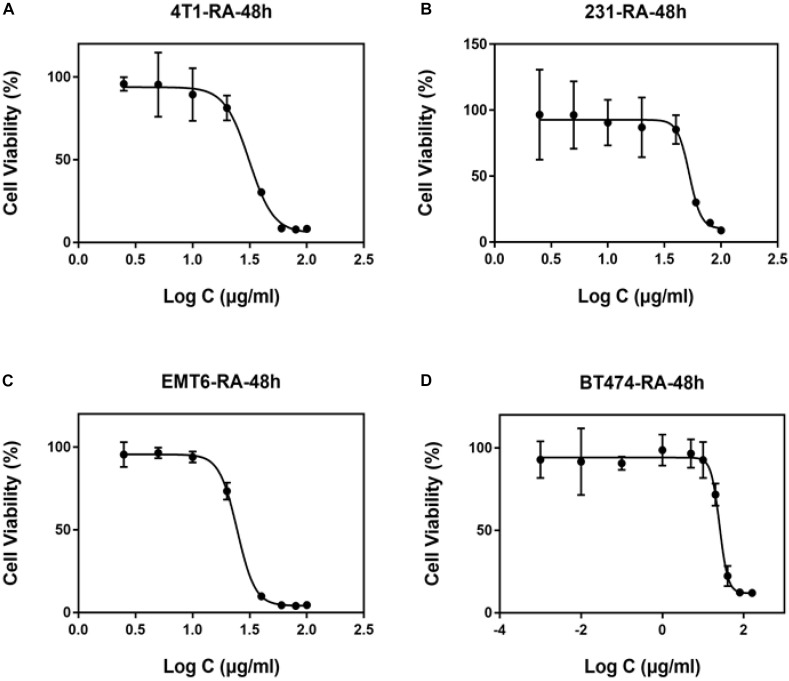
Cytotoxic effects of ATRA on different breast cancer cell lines ATRA toxicity in **(A)** 4T1, **(B)** MDA-MB-231, **(C)** EMT6, and **(D)** BT474 cells.

Next, we evaluated the cytotoxicity of free ATRA and Pluronic-ATRA *in vitro*. We selected different breast cancer cell lines (4T1, MDA-MB-231, EMT6, and BT474) and evaluated cell viability via MTT assay. As shown in Figure 2, ATRA inhibited cell growth and caused cell death in different breast cancer cells. The cytotoxic effects of ATRA against estrogen receptor-negative (ER-) 4T1, EMT6, and MDA-MB-231 cells were less potent, and the half maximal inhibitory concentrations (IC_50_) were relatively high compared to those in estrogen receptor-positive (ER+) BT474 cells. The IC_50_ values in 4T1, MDA-MB-231, and EMT6 cells (Figure 2A–C) were 31.16, 37.58, and 50.48 μg/mL, respectively, while that in BT474 was 25.27 μg/mL (Figure 2D). This suggests that ATRA cytotoxicity depends on the expression level of the RA receptor (RAR) in different breast cancer cells, resulting in different sensitivities to ATRA. This indicates that ATRA may be used as a targeted therapeutic agent for anticancer treatment.

### *In vitro* Cytotoxicity of Pluronic-ATRA in Breast Cancer Cells

**FIGURE 3:**
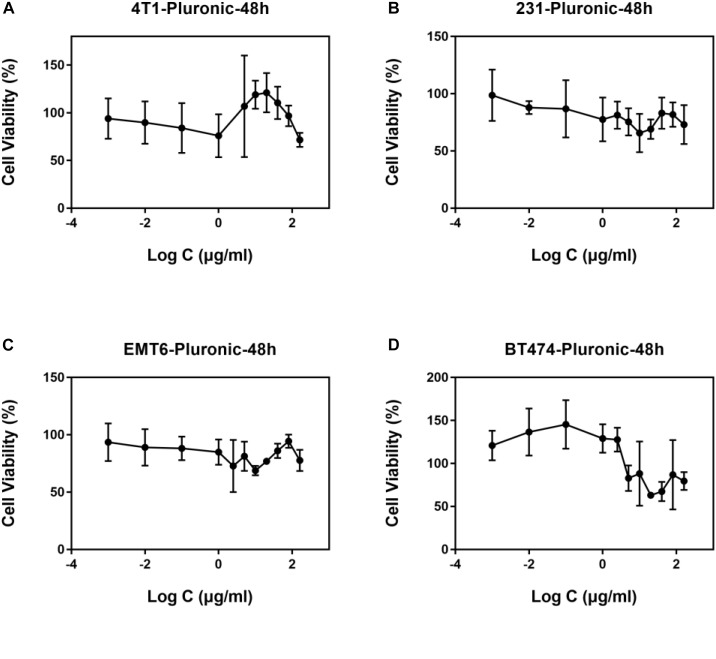
Biocompatibility of Pluronic in breast cancer cell lines **(A)** 4T1, **(B)** MDA-MB-231, **(C)** EMT6, and **(D)** BT474 cells.

After preparing Pluronic-ATRA, we carried out standard Pluronic F127 and Pluronic-ATRA cell toxicity tests before performing further biological experiments. The cytotoxicity of Pluronic alone in 4T1, MDA-MB-231, EMT6, and BT474 cancer cells after 48 h incubation was investigated (Figure 3), and results revealed that Pluronic F127 exhibited negligible cytotoxicity in cancer cells.

**FIGURE 4:**
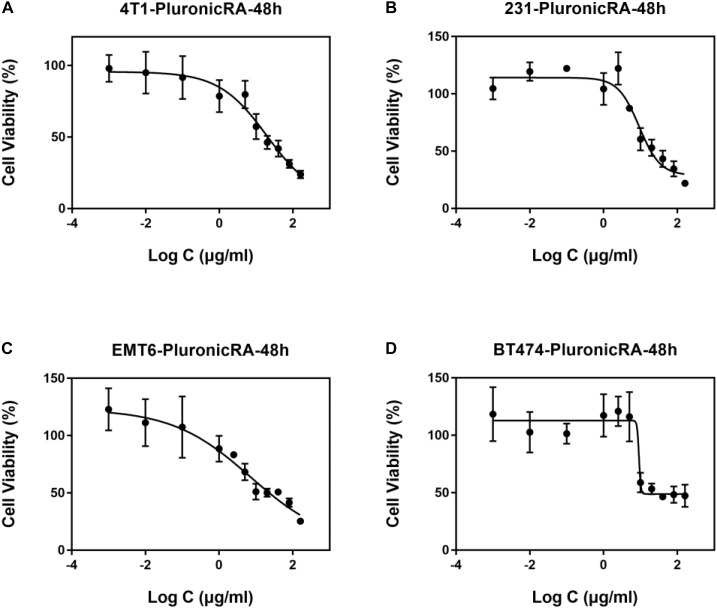
Cytotoxicity of Pluronic-ATRA. Toxic effects of Pluronic-ATRA in **(A)** 4T1, **(B)** MDA-MB-231, **(C)** EMT6, and **(D)** BT474 cells.

The viability of 4T1, MDA-MB-231, EMT6, and BT474 cells after incubation with Pluronic-ATRA for 48 h is shown in Figure 4. Pluronic-ATRA exhibited higher cytotoxicity in all cells than did free ATRA and Pluronic F127. A significant decrease (four to sixfold) was observed in IC_50_ values in MDA-MB-231, 4T1, and EMT6 cells (8.99, 8.57, and 7.08 μg/mL, respectively) compared with those of free ATRA treatment (50.48, 31.19, and 37.58 μg mL, respectively), as shown in Supplementary Table S1. Cytotoxicity was not enhanced in ER+ BT474 cells compared to ER- cells, with IC_50_ values of 9.09 and 25.27 μg/mL, respectively, after treatment with free ATRA. These results prove that Pluronic-ATRA monotherapy enhanced inhibitory effects on breast cancer cells compared to those of free ATRA, and that Pluronic F127 itself showed very limited or even no effect on cells. This suggests that Pluronic-ATRA would be a more effective therapeutic agent than free ATRA and may be used with other drugs in cancer treatment.

### The Combination of Pluronic-ATRA and CDDP Enhanced Cytotoxicity in Breast Cancer Cells

**FIGURE 5:**
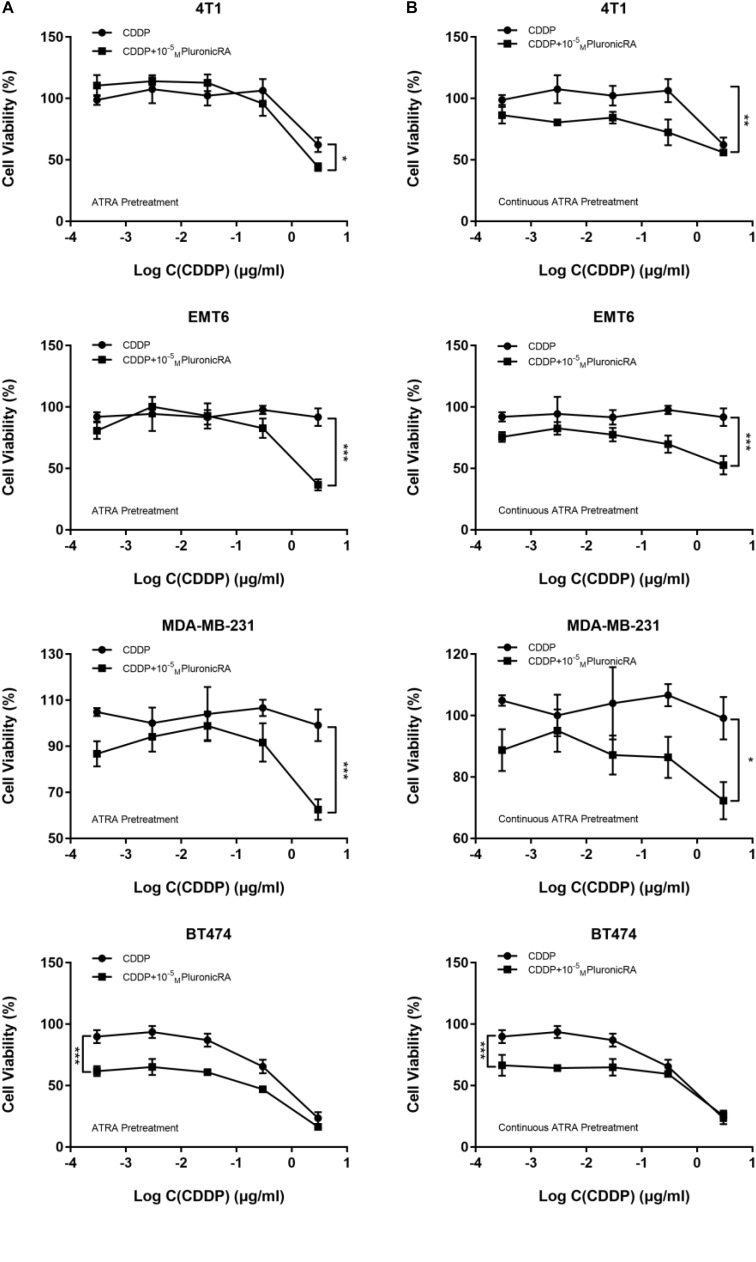
Combined treatment with Pluronic-ATRA and cisplatin (CDDP) on carcinoma after pretreatment with low-dose ATRA. Combination treatment after pretreatment with Pluronic-ATRA **(A)** and continuous Pluronic-ATRA **(B)** with CDDP. ^∗^*P* < 0.05, ^∗∗^*p* < 0.01, and ^∗∗∗^*p* < 0.001.

We further evaluated the *in vitro* anticancer effects of Pluronic-ATRA in combination with CDDP in 4T1, MDA-MB-231, EMT6, and BT474 cells. As depicted in Figure 5A,B, the cytotoxic effects of CDDP in combination with Pluronic-ATRA were enhanced compared to those of CDDP alone. Moreover, pretreatment with a lower concentration of Pluronic-ATRA (10^-5^ M) followed by exposure to CDDP resulted in higher cytotoxicity compared to that after continuous Pluronic-ATRA treatment with CDDP. In addition, ER+ BT474 cells were more sensitive than ER-breast cancer cells. It is speculated that Pluronic-ATRA entered the cells via the RAR and inhibited cell proliferation, further enhancing CDDP efficacy.

### *In vivo* Therapeutic Efficacy

**FIGURE 6:**
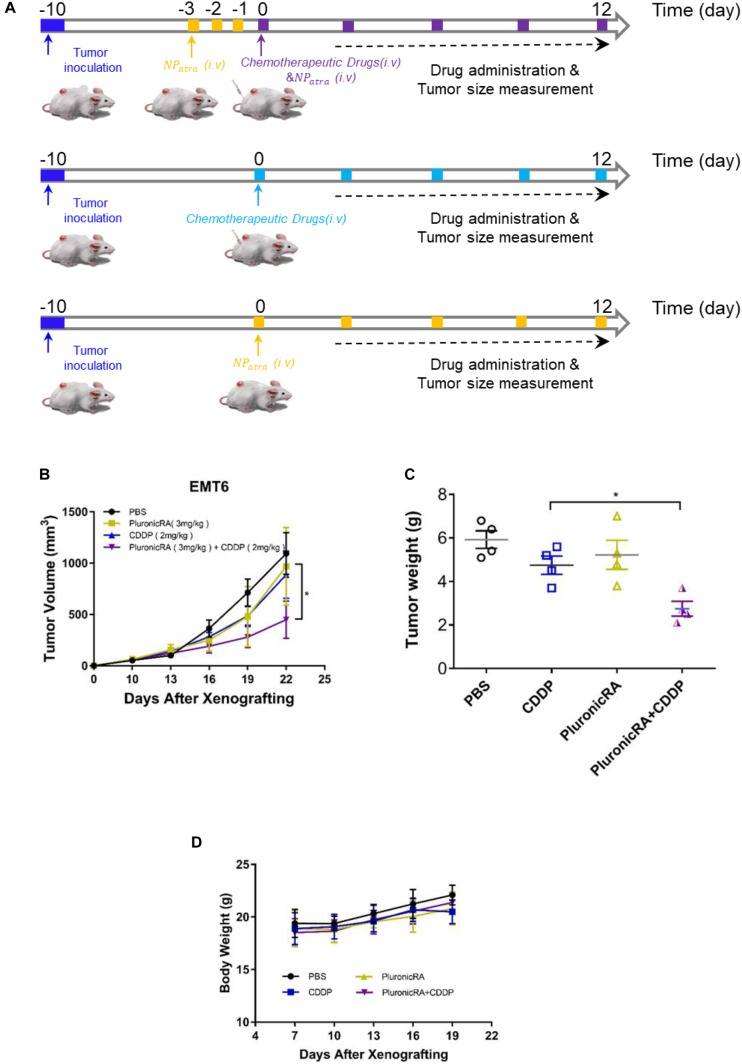
*In vivo* therapeutic study of combination therapy. **(A)** Schematic illustration showing the animal experiment process, **(B)** tumor volume changes in mice receiving phosphate buffered saline (PBS), Pluronic-ATRA, CDDP, and Pluronic-ATRA-CDDP, **(C)** weights of the tumors obtained after the mice were sacrificed, and **(D)** body weights of the mice during the treatment. ^∗^*P* < 0.05.

Following the promising results obtained with combined Pluronic-ATRA-CDDP therapy in cancer cells *in vitro*, we investigated the therapeutic effects of Pluronic-ATRA in solid tumors. To assess whether Pluronic-ATRA-CDDP chemotherapy enhanced the therapeutic effects of CDDP *in vivo*, we established a tumor suppression experiment in female BALB/c mice bearing EMT6 tumors. After randomly dividing the mice into four groups, Pluronic-ATRA and Pluronic-ATRA/CDDP groups were pre-injected with Pluronic-ATRA (3 mg/kg) for 3 days before administering CDDP chemotherapy (Figure 6A). As shown in Figure 6B,C, tumor volume and weight varied among the different treatment groups. As illustrated in Figure 6B, PBS injection showed no inhibitory effects on tumor volume, leading to 14.2-fold volume increases after 21 days. Meanwhile, Pluronic-ATRA and CDDP alone displayed slight inhibitions of tumor growth compared to the control PBS group, although single therapy showed low efficacy. More noticeably, compared with single treatment, the combination of Pluronic-ATRA-CDDP showed the highest tumor growth inhibition and reduced tumor volume and weight, when compared to those in the other treatment groups. These results suggest that the combination of Pluronic-ATRA-CDDP chemotherapy showed higher antitumor efficacy than CDDP chemotherapy and Pluronic-ATRA therapy alone. During treatment, the body weight of mice was measured as an indicator of treatment-induced toxicity. No significant weight loss was observed in any of the treatment groups, suggesting that Pluronic-ATRA-CDDP was safe and had no adverse effects on the mice’s health (Figure 6D), thus making it a safe, novel, and targeted synergistic therapeutic agent for solid tumor therapy.

## Discussion

We designed and conjugated an RA derivative with Pluronic F127 via esterification and successfully prepared Pluronic-ATRA, which showed excellent biocompatibility and high ATRA loading content. In *in vitro* cell experiments, Pluronic-ATRA significantly increased the cytotoxic effects of free ATRA and further enhanced inhibition of different breast cancer cell lines when combined with CDDP. Additionally, tumors were efficiently suppressed by combination therapy of Pluronic-ATRA-CDDP *in vivo*. Therefore, the combination of Pluronic-ATRA and CDDP represents a promising strategy against cancer.

## Ethics Statement

The raw data supporting the conclusions of this manuscript will be made available by the authors, without undue reservation, to any qualified researcher.

## Ethics Statement

All animals received care provided according to the regulations of animal care at University of Science and Technology of China (USTC, Hefei, China). The study was approved by the USTC Animal Care and Use Committee. All animal experimental protocols conformed to the guidelines outlined in the Guide for the Care and Use of Laboratory Animals by the Laboratory Animal Center in USTC.

## Author Contributions

Y-HZ and NY performed the experiments, analyzed the data, and wrote the manuscript. X-FT, MK, H-LL, and NS participated in the experimental design and analyzed the data. L-FH participated in the experimental design and wrote the manuscript.

## Conflict of Interest Statement

H-LL and NS were employed by the company Shandong Liangfu Pharmaceutical Co., Ltd. The remaining authors declare that the research was conducted in the absence of any commercial or financial relationships that could be construed as a potential conflict of interest.
