# Supplementary material for: Synergistic Effect of Retinoic Acid Polymeric Micelles and Prodrug for the Pharmacodynamic Evaluation of Tumor Suppression
Source: Front Pharmacol. 2019 May 16;10:447. doi: 10.3389/fphar.2019.00447 (PMC6531846; doi:10.3389/fphar.2019.00447)
Supplement: Supplementary file 1 [file Data_Sheet_1.PDF]

*Supplementary Material*

# Synergistic Effect of Retinoic Acid Polymeric Micelles and Prodrug for the Pharmacodynamic Evaluation of Tumor Suppression

***Yan-Hua Zhu,‡[1] Ning Ye,‡[1] Xin-Feng Tang,[1] Malik Ihsanullah Khan,[1] Hong-Liang Liu,[2] Ning Shi,[2] Li-Feng Hang, *[1]***

[1] School of Life Sciences, University of Science and Technology of China, Hefei 230027, China

[2] Shandong Liangfu Pharmaceutical Co.,Ltd., Jining 272600, China

*** Correspondence:**

Corresponding Author

hanglf@ustc.edu.cn

**Table S1: $IC_{50}$ of free ATRA and F127-ATRA in breast cancer cell lines**

| Cell Lines / $IC_{50}$ (μg/mL) / Drugs | 4T1 | EMT6 | MDA-MB-231 | BT474 |
|---|---|---|---|---|
| Free ATRA | 31.16 | 50.48 | 37.58 | 25.27 |
| F127-ATRA | 8.57 | 7.08 | 8.99 | 9.09 |

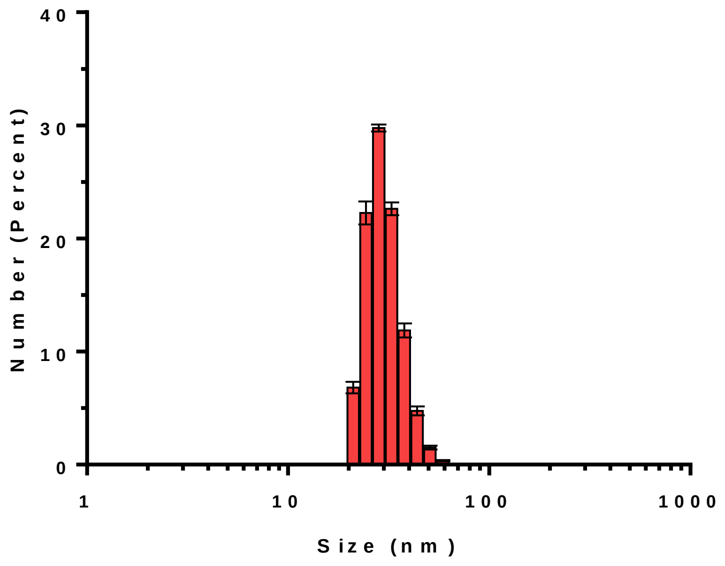


**Supplementary Figure S1. The size distribution of the micelle prodrug.**
